# Effectiveness of six molecular typing methods as epidemiological tools for the study of *Salmonella* isolates in two Colombian regions

**DOI:** 10.14202/vetworld.2019.1998-2006

**Published:** 2019-12-19

**Authors:** Kelly Lozano-Villegas, Roy Rodríguez-Hernández, Iang Rondón-Barragán

**Affiliations:** 1 Research Group in Immunobiology and Pathogenesis, Faculty of Veterinary Medicine and Zootechnics, University of Tolima, Santa Helena Highs, Ibagué 730006299, Colombia; 2 Poultry Research Group, Faculty of Veterinary Medicine, University of Tolima, Santa Helena Highs, Ibagué 730006299, Colombia

**Keywords:** dendrogram, serotyping, typing methods

## Abstract

**Aim:**

The aim of this study was the genotypic characterization of the strains of *Salmonella* spp. isolated from broiler chickens and humans with gastroenteritis from two regions of Colombia, by BOXA1R-polymerase chain reaction (PCR) and random amplification of polymorphic DNA (RAPD)-PCR methods.

**Materials and Methods:**

Forty-nine strains of *Salmonella* were assessed, 15 from poultry farms in Santander region, and 34 from Tolima region isolated from poultry farms (n=24) and the stool samples of people with gastroenteritis (n=10). BOXA1R primers were selected for repetitive element-based PCR (REP-PCR) and five arbitrary primers, namely, GTG 5, OPB 15, OPP 16, OPS 11, and P 1254 were used for RAPD-PCR to generate DNA fingerprints from the isolates. Fingerprint data from each typing method were under composite analysis and the diversity of the data was analyzed by grouping (clustering). The dendrogram was generated by the unweighted group method with analysis of the arithmetic mean based on the Dice similarity coefficient. In addition, Simpson’s index was evaluated to discriminate the power of the methods.

**Results:**

OPP 16 primer and composite analysis proved to be superior compared to other REP-PCR typing methods. The best discriminatory index was observed when GTG 5 (0.92) and OPP 16 (0.85) primers were used alone or combined with RAPD-PCR and BOX-PCR (0.99).

**Conclusion:**

This study indicated that OPP 16 and GTG 5 primers provide suitable molecular typing results for the discrimination of the genetic relationship among *Salmonella* spp. isolates and may be useful for epidemiological studies.

## Introduction

*Salmonella* is a ubiquitous genus of bacteria with global public health concern due to it is the leading cause of foodborne illness accounting for 93.8 million cases and 155,000 deaths per year worldwide [1,2]. Clinical manifestations of salmonellosis vary from self-limiting diarrhea in healthy adults to systemic disease, particularly in susceptible individuals including immunocompromised patients, children, and elderly people [3].

Discrimination of *Salmonella* spp. isolates beyond species level is imperative for effective epidemiological investigation during outbreak events [4]. Serotyping is one of the traditional methods for subspecies typing of *Salmonella* spp. and approximately 2600 serotypes according to the Kauffman–White scheme have been described, considering differences in flagellar (H), capsular (K), and somatic (O) antigens [5]. However, serotyping methods often lack value as an epidemiological tool due to a low discriminatory capacity for strains with identical serotype or similar biochemical characteristics [6]. Molecular subtyping methods have many advantages over traditional methods, such as increased discriminatory power, better standardization, and reproducibility [7]. Molecular typing of *Salmonella* spp. has been employed for screening the origin of common source outbreaks and the identification of relationships among different isolates [8]. Polymerase chain reaction (PCR)-based random amplification of polymorphic DNA (RAPD) and BOX elements can capture variation on a genomic scale as well as determine specific gene variation which is useful for subtyping [9]. RAPD-PCR has demonstrated a high discriminatory potential in the epidemiological studies of closely related bacterial strains including *Salmonella* [10]. In addition, BOXA1R elements [11] are inverted repeated sequences present in a limited number of bacterial species, including *Salmonella*, which allow their subtyping with some limitations [12]. In RAPD, genomic DNA (gDNA) is amplified by PCR with short arbitrary primers to produce distinctive patterns of DNA amplicons, allowing genetic mapping, fingerprinting, and the analysis of interspecific and intraspecific population polymorphisms [13]. Furthermore, RAPD-PCR is a powerful tool for genetic analysis of the phylogenetic relationship among strains for a variety of microorganisms [14].

This study aimed to evaluate the ability of different repetitive element-based PCR (REP-PCR) methods such as BOX-PCR and RAPD-PCR to distinguish between different serotypes of *Salmonella* isolated from poultry and human.

## Materials and Methods

### Ethical approval

No ethical approval required for this study because samples were from Bacterial Strain Collection of the Laboratory of Immunology and Molecular Biology. All the procedures for previous collection of the samples from animals and human were approved by Bioethics Committee of the Central Office of Research from University of Tolima and complied with the guidelines for animal care and use in research and teaching.

### Bacterial strains

A total of 50 *Salmonella* strains were isolated from poultry and fecal samples of patients with gastroenteritis in two regions of Colombia, Tolima, and Santander. Twenty-four isolates were obtained from Tolima’s poultry farms and serotyped as *Salmonella* Paratyphi B. Fifteen isolates were obtained from Santander’s poultry farms and all strains belonged to *S*. Heidelberg serotype. Finally, ten strains from patients with gastroenteritis cases were serotyped as *S*. Newport (n=1), *Salmonella* Enteritidis (n=4), *Salmonella* Braenderup (n=1), *Salmonella* Uganda (n=1), *Salmonella* Typhimurium (n=2), and *Salmonella* Grupensis (n=1) (Table-1). These strains were obtained from the previous studies of the Poultry Research Group of the University of Tolima [15-17].

**Table-1:** Sample ID, source, and locality for 49 *Salmonella* isolates examined in this study.

Sample ID	Serotype	Source	Locality	RAPD					BOX
	
				GTG 5	OPS 11	OPP 16	P 1254	OPB 15	BOXA1R
1	*S.* Heidelberg	Poultry	Santander	-	-	-	-	-	B3
2	*S.* Heidelberg	Poultry	Santander	-	-	-	-	-	B3
3	*S.* Heidelberg	Poultry	Santander	-	-	-	-	-	B3
4	*S.* Heidelberg	Poultry	Santander	-	-	-	-	-	B4
5	*S.* Heidelberg	Poultry	Santander	-	-	-	-	-	B5
6	*S.* Heidelberg	Poultry	Santander	-	-	-	-	-	B6
7	*S.* Heidelberg	Poultry	Santander	-	-	-	-	-	B2
8	*S.* Heidelberg	Poultry	Santander	-	-	-	-	-	B1
9	*S.* Heidelberg	Poultry	Santander	-	-	-	-	-	B6
10	*S.* Heidelberg	Poultry	Santander	-	-	-	-	-	B1
11	*S.* Heidelberg	Poultry	Santander	-	-	-	-	-	B7
12	*S.* Heidelberg	Poultry	Santander	-	-	-	-	-	B7
13	*S.* Heidelberg	Poultry	Santander	-	-	-	-	-	B7
14	*S.* Heidelberg	Poultry	Santander	-	-	-	-	-	B7
15	*S.* Heidelberg	Poultry	Santander	-	-	-	-	-	B8
16	*S.* Paratyphi B	Poultry	Tolima	G4	PP12	PS10	P2	O20	B13
17	*S.* Paratyphi B	Poultry	Tolima	G5	PP12	PS10	P1	O5	B13
18	*S.* Paratyphi B	Poultry	Tolima	G4	PP12	PS10	P1	O5	B17
19	*S.* Paratyphi B	Poultry	Tolima	G3	PP12	PS10	P1	O5	B17
20	*S.* Paratyphi B	Poultry	Tolima	G1	PP12	PS10	P1	O1	B17
21	*S.* Paratyphi B	Poultry	Tolima	G1	PP12	PS10	P2	O3	B17
22	*S.* Paratyphi B	Poultry	Tolima	G1	PP12	PS10	P2	O1	B17
23	*S.* Paratyphi B	Poultry	Tolima	G1	PP12	PS10	P1	O1	B17
24	*S.* Paratyphi B	Poultry	Tolima	G1	PP12	PS10	P1	O9	B17
25	*S.* Paratyphi B	Poultry	Tolima	G7	PP16	PS10	P1	O10	B19
26	*S.* Paratyphi B	Poultry	Tolima	G6	PP9	PS10	P2	O11	B20
27	*S.* Paratyphi B	Poultry	Tolima	G6	PP12	PS10	P2	O1	B19
28	*S.* Paratyphi B	Poultry	Tolima	G8	PP9	PS10	P2	O2	B18
29	*S.* Paratyphi B	Poultry	Tolima	G1	PP9	PS10	P1	O4	B10
30	*S.* Paratyphi B	Poultry	Tolima	G6	PP9	PS8	P1	O10	B10
31	*S.* Paratyphi B	Poultry	Tolima	G7	PP11	PS5	P1	O12	B10
32	*S.* Paratyphi B	Poultry	Tolima	G7	PP11	PS5	P1	O8	B10
33	*S.* Paratyphi B	Poultry	Tolima	G7	PP11	PS5	P3	O14	B23
34	*S.* Paratyphi B	Poultry	Tolima	G7	PP12	PS4	P3	O7	B10
35	*S.* Paratyphi B	Poultry	Tolima	G7	PP12	PS4	P1	O7	B10
36	*S.* Paratyphi B	Poultry	Tolima	G10	PP7	PS9	P3	O16	B10
37	*S.* Paratyphi B	Poultry	Tolima	G9	PP8	PS4	P3	O15	B10
38	*S.* Paratyphi B	Poultry	Tolima	G2	PP8	PS4	P3	O13	B9
39	*S.* Paratyphi B	Poultry	Tolima	G6	PP8	PS4	P1	O13	B9
40	*S.* Newport	Human	Tolima	G11	PP5	PS4	P4	O5	B22
41	*S.* Enteritidis	Human	Tolima	G16	PP1	PS3	P10	O21	B21
42	*S.* Enteritidis	Human	Tolima	G18	PP1	PS3	P10	O19	B21
43	*S.* Enteritidis	Human	Tolima	G19	PP1	PS3	P6	O19	B21
44	*S.* Braenderup	Human	Tolima	G15	PP4	PS2	P9	O6	B9
45	*S.* Uganda	Human	Tolima	G20	PP2	PS6	P10	O5	B16
46	*S.* Enteritidis	Human	Tolima	G17	PP1	PS3	P6	O20	B21
47	*S.* Typhimurium	Human	Tolima	G12	PPP6	PS5	P7	O17	B11
48	*S.* Grupensis	Human	Tolima	G14	PP3	PS1	P8	O18	B14
49	*S.* Typhimurium	Human	Tolima	G13	PP6	PS7	P5	O17	B12

*S.* Heidelberg=*Salmonella* Heidelberg, *S.* Paratyphi=*Salmonella* Paratyphi, *S.* Enteritidis=*Salmonella* Enteritidis, *S.* Braenderup=*Salmonella* Braenderup, *S.* Uganda=*Salmonella* Uganda, *S.* Typhimurium=*Salmonella* Typhimurium, *S.* Grupensis=*Salmonella* Grupensis

### gDNA extraction

gDNA was extracted from fresh colonies using the Invisorb® Spin Universal Kit (Stratec, Germany) following the manufacturer’s instructions. In addition, all isolates were confirmed by PCR through amplification of the *invA* gene (accession number NC 003197.2) using the primers forward 5´-TGAAATTATCGCCACGTTCGGGCAA-3´ and reverse 5´-TCATCGCACCGTCAAAGGAACC-3´ with an amplicon size of 285 bp [17]. *S*. Enteritidis ATCC® 13076 strain (ATCC, USA) was used as a positive control.

### BOX-PCR

The primer BOXA1R 5´-CTACGGCAAGGCG ACGCTGACG-3´ [18] was used for BOX-PCR fingerprinting. The 25 µL reaction mixture contained 5 µL of 5× Colorless GoTaq® Flexi Buffer, 2 µL of deoxynucleoside triphosphate mix, 1 µL of primer, 2 µL of magnesium chloride, 0.5 U of GoTaq® Flexi DNA polymerase (Promega, Madison, USA), and 1 µL of gDNA template. PCR condition included initial denaturation at 95°C for 2 min, followed by 35 cycles of denaturation at 92°C for 1 min, primer annealing at 50°C for 2 min, and extension at 72°C for 8 min, with a final extension at 72°C for 12 min.

### RAPD-PCR

The GTG 5 primer 5´-GTGGTGGTGGTG GTG-3 [19], the OPP 16 5´-CCAAGCTGCC-3´ and OPS11 5´-AGTCGGGTGG-3 primers [20], the P 1254 primer 5´-CC GCA GCCAA-3´ [21], and the primer OPB 15 5´-CCAGG GTGTT-3´ [22] were selected for the RAPD. The PCR was performed in 25 µL volume containing 5 µL of 5× Colorless GoTaq® Flexi Buffer, 2 µL of deoxynucleoside triphosphate mix, 1 µL of primer, 2 µL of magnesium chloride, 0.5 U of GoTaq® Flexi DNA polymerase (Promega, Madison, USA), and 1 µL of gDNA template. PCR condition included initial denaturation at 95°C for 2 min, followed by 35 cycles of denaturation at 92°C for 1 min, annealing at 35°C for 2 min, and extension at 72°C for 5 min, with a final extension at 72°C for 8 min.

### Gel electrophoresis

After PCR amplification, 6 µL of each amplified product was fractionated by electrophoresis using 1% agarose gel (Ultrapure™ Agarose, Thermo Fisher Scientific, USA) in a 1× TBE buffer. The gel was stained with Hydra Green™ (ACT Gene, Piscataway, NJ) and viewed under an ultraviolet transilluminator (Enduro™ GDS, Labnet International, USA). A 1 kb DNA ladder (Thermo Fisher Scientific, USA) was included in each gel as a molecular weight marker.

### Cluster analysis

Gel images were normalized and bands were identified and statistically analyzed using BioNumerics software (version 7.5; Applied Maths, Kortrijk, Belgium). The similarities between DNA fingerprints were calculated with the band-based method of Dice [23]; with ranges from 0 to 1.0, where 1.0 represents 100% of identity (presence and position) for all bands in the two PCR fingerprints being compared. The dendrograms were constructed using the unweighted pair group method with arithmetic averages clustering method.

### Discriminatory index (D)

The discriminatory power (D values) of typing methods was calculated based on Simpson’s index of diversity using the formula described by Hunter and Gaston [24].


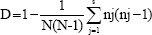


Where D is the discriminatory power, N is the total number of strains unrelated, s is the total number of types described, and j is the number of strains belonging to j type. A value of 1 is highly discriminatory and a value of 0 is not discriminatory.

## Results

### REP-PCR analysis

BOXA1R primers generated fingerprinting patterns for all the isolates examined (Table-1). The multiple DNA fragments showed with BOXA1R primers ranged in sizes between 250 and 2500 bp. No common bands were observed in all 49 *Salmonella* isolated, but 10 sets of isolates showed identical banding.

### RAPD-PCR analysis

RAPD primers used in this study generated fingerprinting patterns for all the isolates except *Salmonella* Heidelberg strains, which were unable to type by any of the RAPD methods (Table-1). Molecular typing of *Salmonella* isolates using the GTG 5, OPB 15, OPP 16, OPS 11, and P 1254 primers generated 9, 9, 6, 4, and 6 numbers of single isolate, respectively, with D values of 0.92, 0.95, 0.85, 0.79, and 0.81, respectively (Table-2). Primer GTG 5 produced bands ranging in size from 250 to 2500 bp; primer OPB 15 produced bands ranging from 300 to 4000 bp; OPP 16 produced bands with molecular weight ranging from 250 to 3500. Bands ranging in size from 300 to 2500 bp were found with primers OPS 11 and P 1254.

**Table-2:** Discriminatory index of BOX-PCR (n=49) and RAPD-PCR (n=34) in genotyping of *Salmonella* isolated.

Genotyping method	Similarity index (%)	Number of clusters	Cluster sizes	Number of single isolates	Discriminatory index
GTG 5	80	4	22,2,2,4	4	0.76
	90	5	8,3,10,2,2	9	0.92
OPP 16	80	5	2,8,2,4,15	3	0.75
	90	6	4,2,3,4,3,12	6	0.85
OPS 11	80	3	4,13,14	3	0.69
	90	5	4,6,4,2,14	4	0.79
P 1254	80	5	19,5,2,2,3	3	0.67
	90	6	13,6,5,2,2,3	6	0.81
OPB 15	80	7	7,6,10,3,2,2,2	2	0.84
	90	9	4,5,3,2,2,2,2,2	9	0.95
BOXA1R	80	6	3,7,5,17,11,4	2	0.80
	90	9	2,5,2,4,12,2,2,11,4	5	0.88

PCR=Polymerase chain reaction, RAPD=Random amplification of polymorphic DNA

### Composite analysis

Composite analysis increased the discriminatory index of *Salmonella* strains by the combination of two different typing methods, presenting D values of up to 0.99. These values were present in the composite analysis using all methods or with the combination of OPP 16, P 1254, and GTG 5 primers. Furthermore, all the combinations for composite analysis were highly discriminatory (D value >0.90). The combination of two separate BOX and RAPD patterns produced clusters with 0.98, 0.97, 0.97, and 0.98 discrimination index values. Likewise, the combination of two separate RAPD fingerprinting patterns produced clusters with 0.96, 0.98, 0.96, and 0.95 discrimination index values (Table-3).

**Table-3:** Composite analysis of different molecular methods.

Composite procedure	Genotyping method	Similarity index (%)	Number of clusters	Cluster sizes	Number of single isolates	Discriminatory index
1	All methods	80	8	2,7,2,4,2,2,2,2	13	0.95
		90	4	2,3,2,2	27	0.99
2	RAPD	80	8	4,2,2,2,7,3,2,2	12	0.94
		90	4	2,2,4,3	25	0.98
3	BOXA1R-GTG 5	80	7	4,2,7,3,3,3,3	11	0.94
		90	4	3,4,2,2	25	0.98
4	BOXA1R-OPP 16	80	9	8,2,2,3,3,2,2,2,4	8	0.93
		90	6	3,5,2,2,2,4	18	0.97
5	BOXA1R-P 1254	80	7	2,6,5,7,2,3,2	9	0.92
		90	7	3,3,2,4,2,2,3	17	0.97
6	BOXA1R-OPB 15	80	7	3,9,2,2,4,3,4	9	0.91
		90	7	2,4,2,2,2,3,2	19	0.98
7	GTG 5-OPP 16	80	8	8,2,3,2,2,3,2,3	11	0.93
		90	5	7,2,2,2,2	21	0.96
8	GTG 5-P 1254	80	6	2,6,3,2,7,4	12	0.93
		90	6	3,2,3,3,2,2	23	0.98
9	GTG 5-OPB 15	80	7	3,3,6,2,2,4,2	14	0.97
		90	4	2,2,6,3	23	0.96
10	OPP 16-P 1254	80	8	4,4,3,6,4,3,2,2	8	0.93
		90	7	3,3,2,6,4,2,2	14	0.95
11	OPP 16-P 1254-GTG 5	80	7	6,3,2,3,4,2,2	14	0.95
		90	6	3,2,2,2,2,2	23	0.99

### Analysis of dendrogram

**Figure-1:**
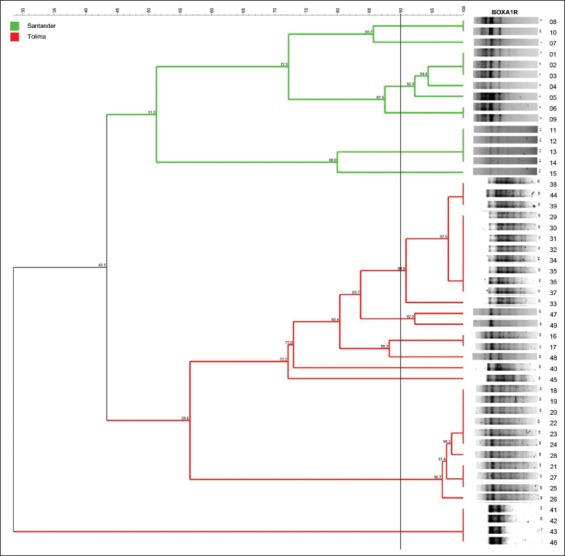
Amplicon profile and phylogenetic tree from BOX-PCR of 50 *Salmonella* Enteritidis fingerprints showing the genetic relatedness of isolates (1-15) obtained from broiler farms in Santander (n=15), isolates (16-39) obtained from broiler farms in Tolima (n=24), and isolates (40-49) obtained from stool samples of people with gastroenteritis; clusters were obtained according to the arbitrary 90% cutoff value for grouping by genotype similarity.

**Figure-2:**
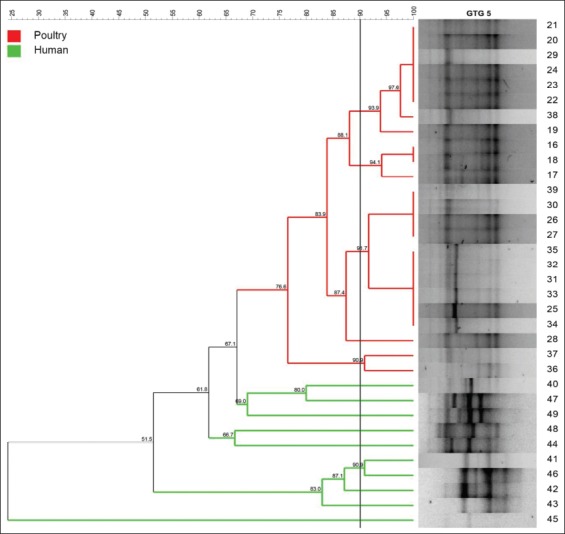
Amplicon profile and phylogenetic tree from GTG 5 of 35 *Salmonella* Enteritidis fingerprints showing the genetic relatedness of isolates (16-39) obtained from broiler farms in Tolima (n=24) and isolates (40-49) obtained from stool samples of people with gastroenteritis; clusters were obtained according to the arbitrary 90% cutoff value for grouping by genotype similarity.

**Figure-3:**
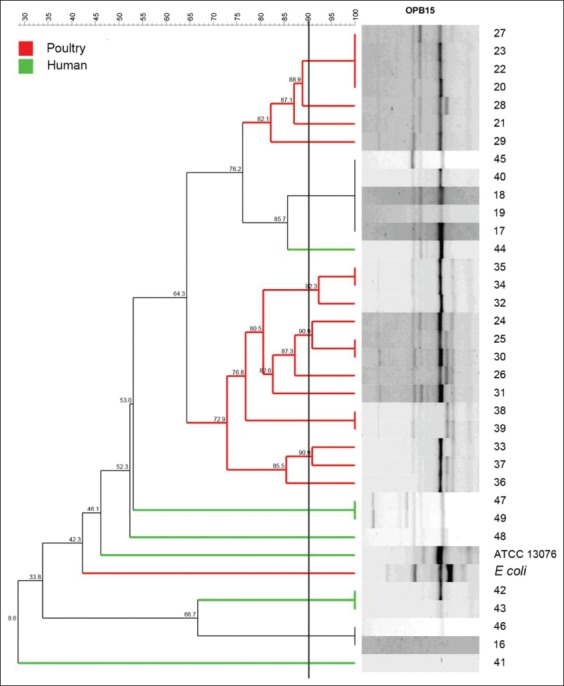
Amplicon profile and phylogenetic tree from OPB 15 of 35 *Salmonella* Enteritidis fingerprints showing the genetic relatedness of isolates (16-39) obtained from broiler farms in Tolima (n=24) and isolates (40-49) obtained from stool samples of people with gastroenteritis; clusters were obtained according to the arbitrary 90% cutoff value for grouping by genotype similarity.

**Figure-4:**
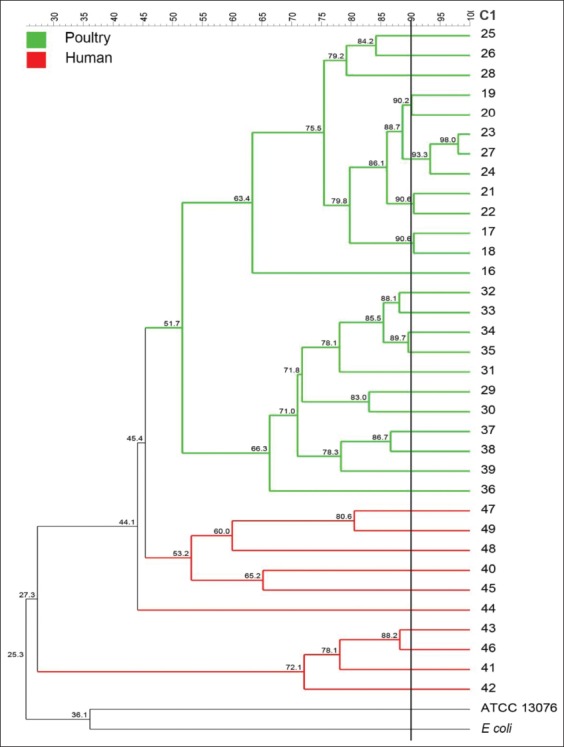
Dendrogram of composite data set based on random amplification of polymorphic DNA and BOX primers. Thirty-five *Salmonella* Enteritidis fingerprints showing the genetic relatedness of isolates (16-39) obtained from broiler farms in Tolima (n=24) and isolates (40-49) obtained from stool samples of people with gastroenteritis; clusters were obtained according to the arbitrary 90% cutoff value for grouping by genotype similarity.

A phylogenetic tree was constructed from each of BOX, GTG 5, OPB 15, and composite PCR amplicon profiles. As shown in Figure-1, BOX grouped the 50 *Salmonella* isolates into nine distinct clusters with 2, 5, 2, 4, 12, 2, 2, 11, and 4 isolates each one. With a similarity level of 90%, five different clusters were distinguished for GTG 5, which demonstrated to be the most suitable molecular typing method for clustering of *Salmonella* isolates genetically related within the same serotype or source (Figure-2). By contrast, OPB 15 primer did not discriminate clusters of isolates with the genetic relationship (Figure-3). Composite 1, which was a combination of RAPD (the GTG 5, OPB 15, OPP 16, OPS 11, and P 1254) patterns and BOX patterns, produced 31 profiles clustering in four clusters and 27 single isolates (Figure-4).

## Discussion

Genotyping methods are useful tools for the retrospective identification of epidemic clones of *Salmonella* and subsequently tracking their dissemination throughout human and animal populations [25] as well as in the design of programs for control of disease, mainly for foodborne pathogens. In this study, RAPD and BOX methods were used as molecular tools to characterize *Salmonella* strains isolated from poultry and stool samples of patients with gastroenteritis (Table-1).

In case of BOX-PCR results of our study (Table-1), similar results were reported by Poonchareon *et al*. [9] who showed that BOX-PCR can differentiate the genetic relationship between *Salmonella* isolates as well as grouping them into different clusters according to their origin. Regarding the amplicons, in this study, BOX-PCR generated bands between 250 and 3000 bp; similar to the previous reports, where amplicons ranged from 400 to 6000 bp [10]. The analysis of fingerprints in the dendrogram showed that BOX-PCR can differentiate the strains according to their geographical distribution, but this method cannot differentiate strains at the serotype level (Figure-1). In the case of the clonal groups, this primer clustered all *S*. Enteritidis isolates in a clonal group. This clustering can be attributed to *S*. Enteritidis since it is a highly clonal serotype [26]. However, BOX-PCR has showed as a discriminatory method for *S*. Enteritidis and *S*. Typhimurium from the different origins [8,22]. In case of *S*. Typhimurium, a diverse serotype in Colombia, it clustered in two single isolates. Since BOXA1R marker discriminated the isolates by geographic origin, it may be useful for the genotyping of *Salmonella* strains.

In RAPD-PCR, amplification of gDNA is based only on one oligonucleotide primer of arbitrary sequence that produced a characteristic spectrum of DNA products of varying sizes [27]. The discriminatory power of this typing method can be enhanced by the use of more than one primer [28]. For this reason, this study incorporated five random primers for differentiating isolates of *Salmonella* species. Genotyping of *Salmonella* spp. using RAPD primers allowed the typing of 34 of 49 strains. *Salmonella* Heidelberg did not generate any band pattern in the RAPD-PCR with any RAPD primer.

GTG 5 primer is a trinucleotide repeat that hybridizes randomly with complementary sequences in the *Salmonella* genome [29] and genotyped different *Salmonella* serotypes (*S*. Bareilly, *S*. Deversoi, *S*. Anatum, *S*. Bredeney, *S*. Gallinarum, *S*. Choleraesuis, *S*. Typhi, *S*. Pullorum, *S*. Montevideo, *S*. Derby, *S*. Weltevreden, *S*. Enteritidis, and *S*. Paratyphi B) [30]. This method proved to be effective for the discrimination of *Salmonella* strains because it can cluster the isolates according to their serotype as well as their source of isolation (Figure-2). The genotyping of *S*. Paratyphi B generated amplicons between approximately 500 and 2500 bp, with amplification profiles characterized by the presence of four clones (Figure-2). Regarding the size of the amplicons, it is consistent with those reported by Rasschaert *et al*. [31] for *S*. Enteritidis, *S*. Braenderup, and *S*. Typhimurium; where amplicons ranged between 500 and 3000 bp.

Based on OPB 15, it was unable to distinguish between *Salmonella* strains based on serotype, origin, or geographical distribution (Figure-3). Although the OPB 15 primer showed the higher discrimination index 0.95 (Table-3), it was not effective for the discrimination among *Salmonella* serotypes such as *S*. Enteritidis and *S*. Paratyphi B or *S*. Paratyphi B, *S*. Uganda, and *Salmonella* Newport (Figure-3); which is different from the results reported by Silva *et al*. [3] who categorize the serotypes of the isolates due to the presence of polymorphic band patterns. Given the characteristics of OPB 15, it was not considered efficient for genotyping of *Salmonella* strains.

Amplicons generated using OPP 16 primer ranged from 500 to 4000 bp. In case of *S*. Paratyphi B, amplicons showed bands from 500 to 3500 bp. The size of the patterns is consistent with that reported for the OPP 16 in *Salmonella* with amplicons between 490 and 5000 bp and the presence of two monomorphic bands (550 and 575 bp) [10,20]. On the other hand, OPS 11 primer elicited bands according to Albufera *et al*. [19] who reported band patterns with size from 400 to 3000 bp.

In this study, P 1254 primer generated amplicons with a larger size (500-4000 bp) than reported by Hashemi and Baghbani-Arani [10], who have reported of bands with sizes ranging from 300 to 3500 bp, from different serotypes of *Salmonella* spp.

In as much as to the discriminatory index of typing methods is an important consideration in epidemiology, particularly in the context of a predominant circulating clone [32]; in our results, we found the discriminatory index was OPB 15 > GTG 5 > BOXA1R > OPB 16 > P 1254 > OPS 11, with *D* values of 0.95, 0.92, 0.88, 0.85, 0.81, and 0.79, respectively (Table-2). This differs from Poonchareon *et al*. [9], who reported the values of 0.99 for BOX and GTG 5 higher than the values reported in this study. In the same way, Hashemi and Baghbani-Arani [10] reported *D* values higher than the values of this study for OPP 16, P 1254, BOX, and OPS 11 of 0.98, 0.98, 0.98, and 0.94, respectively. However, it is important to highlight that the optimal typing method may vary depending on the strain types present in the population, relative clonality of the strains within a collection or differences in the source of the samples (human, animal, or food) [32].

A combination of different typing methods generally increased the discrimination of *Salmonella* spp. (Table-3). However, higher discriminatory power does not always correspond to a more accurate representation of the epidemiologic relationship [33]. This is because the effectiveness of a molecular typing method is not only exclusively determined by the ability to discriminate the unrelated strains but also by the ability to form biological meaningful clustering [34]. Therefore, despite the good results obtained in the discrimination of unrelated strains when was performed the composite analysis, the ability to form specific groupings was evidenced in all combinations, allowing the discrimination of strains concerning their source of isolation (Figure-4). Composite 1 was a combination of RAPD and BOX patterns and based on a similarity level of 90%, it was able to separate isolates into four clusters and 27 single isolates (Table-3). Thus, composite 1 can be efficient for the genotyping of *Salmonella* strains due to its ability to categorize the isolates based on their origin.

## Conclusion

BOXA1R clustering revealed that it could be useful for *Salmonella* genotyping and the development of epidemiological studies since it allows us to obtain data about the genetic relationship of the same locality or source. Therefore, values of the discriminatory index obtained with OPB 15 marker indicated that a high discriminatory index power does not always correspond to a more accurate representation of the epidemiologic relationship. Finally, data obtained in the composite analyses showed that the combination of two different methods increases the discrimination capacity in *Salmonella* spp. isolates.

## Authors’ Contributions

IR and KL conceived and designed the study and performed the statistical analyses. RR collected samples and KL performed the experiments and the laboratory analyses. IR and KL drafted the manuscript and IR revised the manuscript critically. All authors read and approved the final manuscript.
